# Taste receptors affect male reproduction by influencing steroid synthesis

**DOI:** 10.3389/fcell.2022.956981

**Published:** 2022-08-11

**Authors:** Wenjiao Liu, Ting Gong, Fangxiong Shi, Houqiang Xu, Xiang Chen

**Affiliations:** ^1^ Key Laboratory of Animal Genetics, Breeding and Reproduction in the Plateau Mountainous Region, Ministry of Education, Guizhou University, Guiyang, China; ^2^ Guizhou Provincial Key Laboratory of Animal Genetics, Breeding and Reproduction, Guizhou University, Guiyang, China; ^3^ College of Animal Science, Guizhou University, Guiyang, China; ^4^ College of Animal Science and Technology, Nanjing Agricultural University, Nanjing, China

**Keywords:** taste receptors, male reproduction, steroid hormones, spermatogenesis, machanism

## Abstract

For the male genetic materials to reach and fertilize the egg, spermatozoa must contend with numerous environmental changes in a complex and highly sophisticated process from generation in the testis, and maturation in the epididymis to capacitation and fertilization. Taste is an ancient chemical sense that has an essential role in the animal’s response to carbohydrates in the external environment and is involved in the body’s energy perception. In recent years, numerous studies have confirmed that taste signaling factors (taste receptor families 1, 2 and their downstream molecules, Gα and PLCβ2) are distributed in testes and epididymis tissues outside the oral cavity. Their functions are directly linked to spermatogenesis, maturation, and fertilization, which are potential targets for regulating male reproduction. However, the specific signaling mechanisms of the taste receptors during these processes remain unknown. Herein, we review published literature and experimental results from our group to establish the underlying signaling mechanism in which the taste receptor factors influence testosterone synthesis in the male reproduction.

## Introduction

The rapid spread of the COVID-19 disease quickly evolved into a global pandemic. As a result, knowledge of the COVID-19 symptoms in patients is key in the COVID-19 treatment. Among these symptoms is the gustatory dysfunction present in 38.2% of 8438 patients from 13 countries and regions infected with COVID-19 (Agyeman et al., 2020). In addition, COVID-19 patients have characteristic pathological changes in testicular histology, including the varying degree of damage to the seminiferous tubules, a significant decrease in Leydig cells, and severe injury to Sertoli cells (Yang et al., 2020). COVID-19 infection also down-regulates five proteins associated with cholesterol synthesis in the testicular tissue, lowering the cholesterol levels, a precursor for steroid hormone synthesis (Nie et al., 2021). Recent studies have established that besides the tongue, taste receptors are also expressed in the digestive tract, liver, respiratory tract, ovaries, and testes (Damak et al., 2003; Li et al., 2005; Yang et al., 2021). However, the association between taste disturbance and testicular lesions in COVID-19 patients and whether the two are linked through taste receptors remains unknown.

Taste receptors are sensory receptors in the oral cavity expressed in type II taste cells in the oral taste buds (Lee et al., 2017; Ryo et al., 2017). Taste receptors (T1Rs, T2Rs) perceive sweet (T1R2+T1R3), umami (T1R1+T1R3), and bitter (T2Rs) tastes, which they signal through their associated signaling molecules such as α-gustducin (Gα), and phospholipase C subunit 2 (PLCβ2) (Fehr et al., 2007; Nobuhumi et al., 2015; Roper and Chaudhari, 2017; Tian et al., 2019). However, recent studies have found that these taste receptors are also expressed in tissues other than the tongue, such as the digestive tract, liver, respiratory tract, ovaries, and testes (Damak et al., 2003; Li et al., 2005; Yang et al., 2021). Given the importance of reproduction, an increasing number of researchers have focused on the relationship between taste receptors and mammalian testes. Using knockout models, Mosinger et al. established that the double knockout (*Tas1r3* and *Gnat3*) male mice had inactive epididymal sperm and testicular lesions. Besides, the double knockout female mice were fertile but the male mice were sterile (Mosinger et al., 2013). Further investigation on the molecular mechanism of specific male sterility due to taste receptor deletion in mice, researchers found that a selective blockade of the *Tas1r3* gene affecting sperm development and maturation leads to male sterility. However, the male mice could regain normal fertility after withdrawing the inhibitors selectively blocking the *Tas1r3* gene (Mosinger et al., 2013). To the best of our knowledge, male reproduction is regulated by sex hormones, and we hypothesize that the mechanism is due to the changes in steroid hormones. Evidence to support this hypothesis is that, *in vivo*, our study found that T1r3 activation by sodium saccharin increased the expression of steroid-related factors in mice, and we observed similar phenomena in both female and male rats (Gong et al., 2016a; Jiang et al., 2018).

The testis is the male reproductive organ responsible for sperm production and androgen secretion. Sperm production occurs in the seminiferous tubules, while androgen secretion is mediated by Leydig cells (Staub and Johnson, 2018). Spermatogenesis is a complex developmental process that ensures the formation of millions of spermatozoa per day through mitosis, meiosis and complex morphological changes in spermatogonia (Linn et al., 2021). Various testicular somatic cells play a role in spermatogenesis, including Sertoli cells, which provide nutrition and protection to developing sperms, and Leydig cells, which secrete androgen (Neto et al., 2016).

**FIGURE 1:**
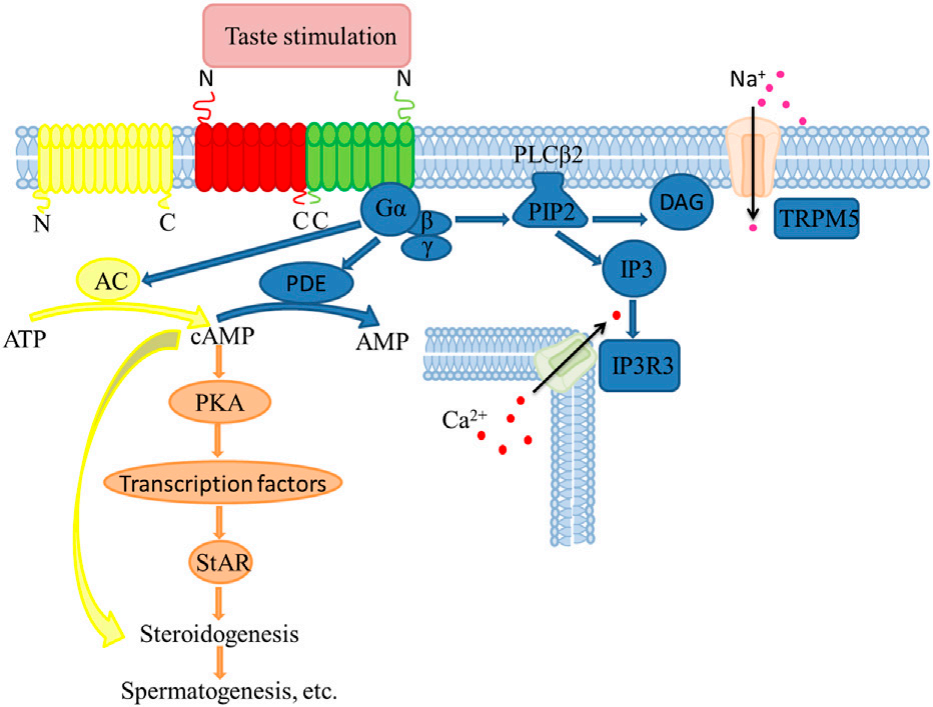
Molecular mechanisms in steroid synthesis regulated by taste receptors in testicular Leydig cells.

T1R3 and its downstream protein Gα in late spermatogenic cells and Leydig cells have been observed in mice and pigs (Spinaci et al., 2014; Gong et al., 2016b; Gong et al., 2021). Besides, T2Rs has ectopic expression during spermatogenesis in human (Governini et al., 2020). Besides, T1r1 and T1r3 are present in the spermatozoa during the immotile to motile phases when spermatozoa are transported to the epididymis for maturation and storage (Frolikova et al., 2020). Moreover, T2Rs are expressed in cumulus and granulosa cells, essential for oocyte development and fertilization (Semplici et al., 2021). T2Rs are also highly expressed in the mouse haploid sperm cells, where the sperm cells respond to bitter-tasting substances possibly through Ca^2+^ (Xu et al., 2013). In addition, the epididymal T1R1 and T1R3 are expressed in the epithelium cells and epididymal spermatozoa in a segment-specific manner (Meng et al., 2020). Sperms undergo capacitation and acrosomal secretion to complete fertilization, which regulated by Ca^2+^ and cyclic adenosine monophosphate (cAMP) signals. However, Ca^2+^ and cAMP concentrations are changed by the activation or absence of *Tas1r1/Tas1r3* (Meyer et al., 2012). Overall, taste receptors are highly expressed during the postnatal development of male reproductive organs, thus, they may be involved in regulating sperm production, maturation, storage and fertilization, all of which are regulated by sex hormones. Therefore, this review synthesizes evidence of the influence of taste receptors on spermatogenesis, maturation, storage and fertilization by regulating steroid synthesis and an outlook on the corresponding signaling pathways (Figure 1).

### Testosterone synthesis

The classical hypothalamus-pituitary portal system regulates the synthesis of testosterone. Precisely, gonadotropin-releasing hormone (GnRH) is released from the hypothalamus, which acts on the pituitary gland, releasing follicle-stimulating hormone (FSH) and luteinizing hormone (LH). Next, LH binds receptors on Leydig cells membranes, activating adenylate cyclase (AC) and promoting intracellular cAMP production. It is well known that cAMP and protein kinase A (PKA) are crucial synthesizing testicular steroids. For example, in our previous experiments, mice after saccharin sodium injection caused an elevation in taste signaling factors (T1r3, Gα) and also detected an elevation in cAMP concentration, which in turn increased the expression of steroidogenic enzymes (StAR, CYP11A1, 3β-HSD and CYP17A1 (Gong et al., 2021). In addition, the taste receptor T1r1 is linked to cAMP (Meyer et al., 2012). Thus, cAMP activates PKA inducing the phosphorylation of cAMP-response element-binding protein (CREB). Activated CREB up-regulates steroidogenic acute regulatory protein (StAR), which accelerates cholesterol transport from the outer mitochondrial membrane to the inner mitochondrial membrane by binding the translocator protein (TSPO) on the outer mitochondrial membrane. Subsequently, cholesterol is converted to pregnenolone by cytochrome P450 cholesterol side-chain lyase (CYP11A1) in the inner mitochondrial membrane, which is transported to the endoplasmic reticulum, where it is converted to pregnenolone by 3β-hydroxysteroid dehydrogenase (3β-HSD). Progesterone is converted to androstenedione in action catalyzed by CYP17A1. Finally, 17β-hydroxysteroid dehydrogenase (17β-HSD) catalyzes the formation of testosterone from androstenedione (Riccetti et al., 2017).

### Spermatogenesis

In mammals, spermatogenesis is a long physiological process consisting of four stages. First, spermatogonia are transformed into primary spermatocytes through mitosis and secondary spermatocytes after the first phase of meiosis. The haploid spermatozoa are then developed in the second phase of meiosis. Finally, round spermatids are transformed into spermatozoa through complex morphological changes, including chromatin remodeling and compaction (Guercio et al., 2020). The formed spermatozoa are transported to the epididymis for maturation and storage, where they gaining motility and acrosomal function for subsequent capacitation and fertilization in the female reproductive tract (Mahe et al., 2021). Spermatogenesis is regulated by hormones, local regulators, and miRNAs, which ensures the correct genetic and epigenetic information, is transmitted to the offspring.

### Effect of hormones on spermatogenesis

Spermatogenesis is mediated by the endocrine and testicular autocrine/paracrine factors, such as FSH, LH, and testosterone in the Leydig and Sertoli cells (Roper and Chaudhari, 2017; Lara et al., 2020; Sawaied et al., 2020). Other hormones are also involved in regulating spermatogenesis, such as insulin and thyroid hormone (de Kretser et al., 1998). The nerve axis initiates spermatogenesis in the hypothalamus, which triggers the release of GnRH, GnRH acts on the pituitary gland releasing FSH and LH. Each substance has its specific physiological role in this complex process. FSH promotes the growth and spermatogenesis of the seminiferous tubules the transformation of primary spermatocytes into secondary spermatocytes (Spaliviero et al., 2004). On the other hand, LH mediates steroid production by stimulating Leydig cells and promoting the completion of meiosis and morphological changes in sperm. Leydig cells release androgen, which regulates the function of myoid cells including the secretion of active substances that regulate the function of Sertoli cells affecting spermatogenesis of testis and achieving local network regulation of the spermatogenic environment (Li et al., 2020; Pan et al., 2020). Taste receptors are present in spermatogenic cells during spermatogenesis. They reduce T1r3 expression in *Tas1r3* knockout mice and mice fed on high doses of sodium saccharin, reducing sperm viability and causing abnormal sperm morphology. Decrease T1r3 expression subsequently reduced the testosterone and cAMP levels. However, sodium saccharin injection into mouse testes activates T1r3 and Gα while the steroid synthase, testosterone and cAMP levels significantly increase. Nonetheless, the effects and mechanisms of taste receptors during spermatogenesis remain unclear, thus, more research on the influence of taste receptors on spermatogenesis is crucial.

Androgen receptor (AR) regulates spermatogenesis by modulating Ca^2+^ concentrations and downstream protein kinases during meiosis in germ cells. At the same time, cAMP response progenitor regulators control the expression of critical genes such as CREB following meiosis in germ cells. For example, the cAMP response element modulator (CREM), a major transcription factor regulated by cAMP, and spermatogenesis is wholly blocked in *Crem*-deficient male (Blendy et al., 1996; Nantel et al., 1996). *Crem* knockout mice exhibit similar morphological characteristics to our mice fed on high-dose sodium saccharin, while *protamine 1* (*Prm1*), *transition protein 1* (*Tnp1*) and other CREM-regulated genes are down-regulated in *Tas1r3*/*Gnat three* double knockout mice (Blendy et al., 1996; Nantel et al., 1996; Mosinger et al., 2013; Gong et al., 2016a). Besides, TATA and TATA-box binding protein (TBP) activates the spermatogenesis process (Jing et al., 2016; Wu et al., 2016; Murovets et al., 2019). These reports provide insights to explore the mechanism of spermatogenesis further.

### Signal transduction of taste receptors

Sweet, umami and bitter G protein-coupled receptor taste families conduct signal transduction functions through a similar pathway in type II taste bud cells (Liman et al., 2014). The sweeteners and umami agents bind their corresponding receptors, activating the heterotrimer G protein α-gustducin is activated, which releases the Gβγ subunit (a trimeric G protein composed of α-gustducin and a complex consisting of Gβ and Gγ), stimulating PLCβ2 activation. PLCβ2 hydrolyzes the membrane lipid phosphatidylinositol 4, 5-bisphosphate (PIP2) releasing inositol 1, 4, 5-triphosphate (IP3) and diacylglycerol (DAG). The IP3 messenger opens the type 3 ion channel of IP3 receptors (IP3R3) in the endoplasmic reticulum, which initiates the release of Ca^2+^ stored in the endoplasmic reticulum into the cytoplasm. This activates the Ca^2+^-dependent univalent selective cation channel-transient receptor potential channel M5 (TRPM5), depolarizing cytomembranes, which generates an action potential. Simultaneously, α-gustducin activates phosphodiesterase (PDE), which catalyzes the hydrolysis of cAMP to adenosine monophosphate (AMP) (Yan et al., 2001; Zhang et al., 2007; Kinnamon, 2012). Activation of bitter receptors initiates a similar signaling cascade as sweet and umami receptors. However, the bitter taste receptors mainly activate the Ca^2^⁺ signaling pathway and reduce the cAMP level. For example, the cytosolic Ca^2^⁺ and cAMP levels in *Tas1r1*-deficient sperm are significantly elevated (Meyer et al., 2012). In addition, the expression of *Tas1r3* is associated with regulating insulin secretion through apoptosis (Murovets et al., 2019).

### Role of taste receptors in the regulation of male reproduction via sex hormones

Genetic variation in taste receptors regulates male fertility. Given the diversity of taste receptors identified in different species, taste receptors may play different physiological roles in different species through different signaling pathways (Li et al., 2010; Zhao et al., 2010; Jiang et al., 2012). Recent advances show that single nucleotide polymorphisms (SNPs) of homozygous carriers of the G allele of *TAS2R14*-rs3741843 of taste receptor genes are linked to a decreased sperm progressive motility than that in the homozygous carriers. Moreover, the SNPs of homozygous carriers of the T allele of *TAS2R3*-rs11763979 have fewer normal acrosomes than the heterozygous and homozygous carriers of the G allele. The A/G heterozygosis in the SNP of *TAS1R2*-rs4920566 is associated with a decreased number of sperm cells compared to the homozygous carriers of the A allele (Gentiluomo et al., 2017). However, there is no significant difference in the SNP of *TAS2R38* between infertile and fertile men (Siasi and Aleyasin., 2016). Besides, the *TAS1Rs* polymorphisms are linked to food intake, overweight and gastric cancer in humans. For example, the SNP of T allele of *TAS1R1*-rs4908932 SNPs caused increased birth weights compared to the GG homozygotes (Farinella et al., 2021) Table 1.

There exists a relationship between age-related regulation of testosterone synthesis and the expression patterns of T1r3 and Gα at different developmental stages in mice may provide us with a new research idea. For example, in mice treated with a high dose of saccharin sodium, T1r3 and Gα expression in the testis, and the expression of StAR, CYP11Al, 3β-HSD and 17β-HSD are significantly decreased, which corresponds to decline sperm quality and impaired testicular morphology. Interestingly, T1r3 also mediates testicular steroid synthesis in mice by increasing the cAMP levels (Gong et al., 2016a; Gong et al., 2016b). This is consistent with Meyer’s findings, which elaborate on the mechanism of steroidogenesis and spermatogenesis *via* a taste receptors-mediated signal (Meyer et al., 2012).

The influence of taste receptors on male reproduction goes beyond spermatogenesis to fertilization. Specifically, spontaneous acrosomal reaction is significantly increased in *Tas1r1*-deficient mice, and Ca^2+^ levels is significantly higher in the cytoplasm of freshly isolated sperm than in wild-type sperm. In addition, cAMP concentrations were significantly elevated in *Tas1r1*-deficient epididymal spermatozoa. Since Ca^2+^ and cAMP control the basic processes during continuous fertilization, the effect of *Tas1r1* on fertilization is predictable (Meyer et al., 2012). The acrosome response of spermatozoa occurs in the female reproductive tract, where l-glutamate is present as a physiological ligand for T1r3. *In vitro* chemotaxis experiments showed that acrosome responsive spermatozoa are significantly attracted to l-glutamate, which provides a new perspective to resolve the complex fertilization process (Frolikova et al., 2020).

Taste receptors also play a role in the reproductive regulation of females. For example, cAMP regulates both spermatogenesis and fertilization. For example, progesterone regulates sperm fertilization through the cAMP-PKA signaling pathway (Jiang et al., 2020). Although no changes in sex hormones (testosterone and estradiol) were observed in Saccharin sodium and rebaudioside A-fed male guinea pigs in another study, high doses of high saccharin sodium caused damage to testicular and epididymal morphology (Shen and Li, 2021). Furthermore, our recent project found that taste receptors (T1r2, T2r31) and their downstream signaling molecules (Plcb2) and heterotrimeric G proteins (G protein subunit α-gustducin 3, Gnat3; and G protein subunit beta 3, Gnb3) expressed in the corpus luteum of rats affect apoptosis by activating NO/cGMP signaling (reducs the cAMP level). In contrast, activation of the taste receptors reduced the expression of steroid hormone synthase, leading to lower progesterone levels. (Jiang et al., 2021). Similarly, feeding sodium saccharin to female rats caused steroid changes and apoptosis of oocyte and granulosa cells (Kavita et al., 2019). From the available evidence, it appears that the same sweetener can bind to different taste receptors in different species thus causing different effects on steroid hormones. However, in general, the activation of these taste receptors is associated with cAMP, steroid synthase and steroid hormones. Another of our previous results showed that activation of T1r3 in female rats following saccharin sodium treatment caused an abnormal increase in the estrus cycles, ovarian cysts, and serum progesterone levels, all of which were associated with steroids, which was confirmed by the detection of steroid hormone-producing factors (Jiang et al., 2018). Taste receptors affect male and female reproduction differently, perhaps due to differences in taste sensitivity between the two, with testosterone and estrogen modulating the taste-directed behavior and preferences (Martin and Sollars, 2017; Dahir et al., 2021).

Although the relationship between taste receptors and hormones has been demonstrated, the mechanism by which the taste receptors influence spermatogenesis remains unclear. Besides, the current experiments assessing the influence of taste receptors on reproduction have mostly focused on rats and mice model animals, which lack a broad spectrum. Overall, the taste receptors regulate the male reproductive activity by affecting steroid hormone synthesis, mediated through the cAMP signaling pathway, thereby affecting spermatogenesis (Li, 2013; Luddi et al., 2019) (Figure 1).

## Conclusion

The non-taste function of the taste signaling molecules in the testis is associated with steroidogenesis-related factors and intracellular cAMP level. Futher work for clarifying the regulation mechanism of taste receptors in testicular Leydig cells through cAMP-mediated steroidogenic pathway, would help uncover the role of taste receptors in regulation male reproduction.

**TABLE 1:** Related literature on the effect of taste receptors in male reproduction.

Common name	Publication time	Species	Results
Fehr J	2007	*Mouse and rat*	The expression of α-gustducin is highest in differentiated spermatozoa, and it was mainly located in mitochondria of sperm and axoneme
Meyer D	2012	*Mouse*	Deletion of *Tas1r1* gene, abnormal spermatogenesis↓, concentration of Ca2+ and cAMP↑, acrosome reaction↑
Li F	2013	*None*	Reviewed the research progress of taste receptors in spermatogenesis
Mosinger B	2013	*Mouse*	Deletion of *Tas1r3* and *Gnat3*, abnormal sperm↑ and selective blocking *Tas1r3* lead to infertility in male mice
Gong T	2016	*Mouse*	The expression patterns of T1r3 and its associated heterotrimeric Gα in the testis are the same. T1r3 and Gα are highly expressed in the Leydig cells and elongated spermatids after puberty
Gong T	2016	*Mouse*	Taste signaling molecules (T1r3, Gα) activated by sodium saccharin, steroid synthase ↑, and cAMP ↑
Gentiluomo M	2017	*Human*	The genetic homozygosis of *TAS2R14-* rs3741843, abnormal sperm↑, while the genetic homozygosis of *TAS2R3-*rs11763979 and normal acrosome↓
Jiang J	2018	*Rat*	Saccharin sodium-treated and steroidogenesis-related factors ↑ and progesterone↑
Luddi A	2019	*None*	Reviewed the mechanism of taste receptors in male reproduction
Frolikova M	2020	*Mouse*	Selectively blocked *mTas1r3*, chemotaxis of spermatozoa ↓
Governini L	2020	*Human*	*TAS2R14* is the most frequently expressed bitterness receptor in testes and spermatozoa
Farinella R	2021	*Human*	The genetic homozygosis of the *TAS1R1-* rs4908932, birth weight↑
Jiang J	2021	*Rat*	Taste receptors (T1r2*,* T2r31) areactivated by sodium saccharin, steroid synthase ↓ and progesterone ↓
Gong T	2021	*Congjiang Xiang pig*	T1R3 and PLCβ2 are strongly expressed in the cytoplasm of elongated spermatids and interstitial cells, T1R3 and PLCβ2 are highest during puberty

*Tas1r1*, taste receptor type 1 subunit 1, cAMP, cyclic Adenosine monophosphate, *Tas1r3*, taste receptor type 1 subunit 3, Gα (*Gnat3*), G protein α-subunit, T1r3, taste receptor type 1 subunit 3, *TAS2R14*, taste receptor type 2 subunit 14, *TAS2R3*, taste receptor type 2 subunit 3, T1r2, taste receptor type 1 subunit 2*,* T2r31, taste receptor type 2 subunit 31, PLCβ2, phospholipase Cβ2, SNPs: single nucleotide polymorphisms, T1R1, taste receptor type 1 subunit 1, ↑, positive regulation; ↓: negative regulation.
